# High Defibrillation Threshold: The Science, Signs and Solutions

**Published:** 2010-01-07

**Authors:** Sony Jacob, Victorio Pidlaoan, Jaspreet Singh, Aditya Bharadwaj, Mehul B Patel, Antonio Carrillo

**Affiliations:** 1 Cardiac Experimental Electrophysiology laboratory, Division of Cardiology / Electrophysiology, Department of Internal medicine; 2 Wayne State University School of Medicine, Detroit, Michigan, USA; 3 Department of Biomedical Engineering, School of Engineering, Wayne State University, Detroit, Michigan, USA

**Keywords:** Defibrillation threshold testing, DFT, implantable cardioverter defibrillator

## Abstract

Defibrillation threshold (DFT) testing has traditionally been an integral part of implantable cardioverter defibrillator (ICD) implantation. With the increasing number of patients receiving ICDs, physicians are encountering high DFT more often than before. Tackling the problem of high DFT, warrants an in-depth understanding of the science of defibrillation including the key electrophysiological concepts and the underlying molecular mechanisms.  Numerous factors have been implicated in the causation of high DFT. Due consideration to the past medical history, pharmacotherapy, laboratory data and cardiac imaging, help in assessing the pre-procedural risk for occurrence of high DFT. Drugs, procedural changes, type and location of ICD lead system are some of the key players in predicting DFT during implantation. In the event of encountering an unacceptably high DFT, we recommend to follow a step-wise algorithm. Ruling out procedural complications like pneumothorax and tamponade is imperative before embarking on a search for potentially reversible clinical or metabolic derangements. Finally, if these attempts fail, the electrophysiologist must choose from a wide range of options for device adjustment and system modification. Although this review article is meant to be a treatise on the science, signs and solutions for high DFT, it is bound by limitations of space and scope of the article.

## Defibrillation Threshold - an Epidemiological Perspective

Over the past decade, Implantable Cardioverter Defibrillators (ICD) have become the standard of care for patients at risk for sudden cardiac death [1,2]. ICD implantation has been shown to reduce absolute mortality by 8% in primary prevention recipients [3] vis-a-vis a reduction of 7% in secondary prevention recipients [4].  Defibrillation Threshold (DFT) testing has traditionally been part of ICD implantation [5]. DFT is the minimum amount of energy required to reliably defibrillate the heart and represents one of the points of a patient's probability-of-success curve. It is determined by inducing ventricular arrhythmias often under deep sedation and allowing the ICD to detect and deliver therapy to terminate the arrhythmia. Although there have been reports suggesting that DFT testing does not predict survival or improve clinical outcomes in ICD recipients, there is no clear consensus about steering away from this convention [6,7].

High DFT is defined as an absolute value of shock energy >25Joules (J) or a safety margin of < 10J below the maximum output of the device. This is assessed by two successful shocks of same strength [8] and the reported incidence of high DFT is from 2 to 24%. Russo et al reported a 6.2% prevalence of high DFT (n=1139) [9] which was replicated in a separate study by Osswald and colleagues in a larger population (n= 2803) [10]. Although small in number, patients with high DFT pose a risk of sudden cardiac death. A better understanding of the science of defibrillation, available technological options, clinical signs and the solutions for management is crucial and is described in this article.

## The Science

**Figure 1:**
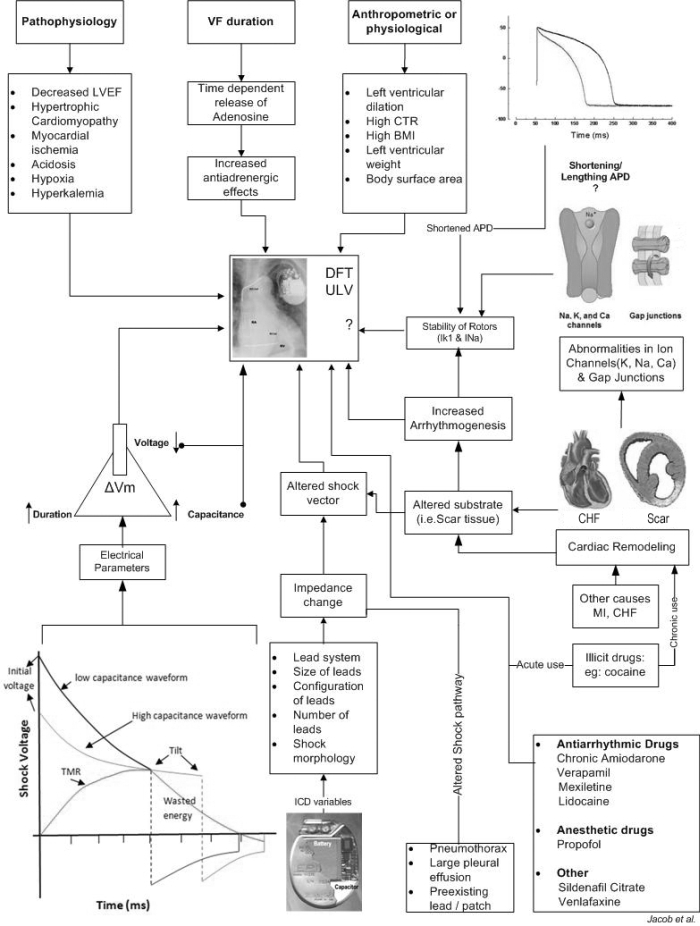
Flowchart describing the interplay of electrical, electrophysiological, molecular and anatomical factors that favours high Defibrillation threshold (DFT). Key electrical parameters that influence the DFT are voltage and the duration it is being applied. The device related factors are the capacitance of the device and the impedance of the coil-tissue composite. The shock voltage - duration graph shows the relationship of the capacitance and voltage in relationship to the transmembrane response (TMR). 'Wasted energy' is the component of the delivered energy which is counterproductive when the duration of application is beyond the peak TMR, particularly with high capacitance energy devices. (Note the inverse relationship of the initial voltage and the capacitance of the ICD). ICD's (implantable cardioverter defibrillator) programmable features, if not appropriately programmed will alter the shock vector and thus can influence the DFT. Antiarrhythmics and other drugs can directly and indirectly affect the DFT. Cardiac pathology like MI or medications can affect the ionic mechanisms responsible for the membrane stability. This can increase the arrhythmogenic potential and can influence the DFT. Several mechanisms are still investigational or has conflicting study results and hence marked with '?'. Other pathophysiological and anthropometric factors are also included for completion.  (LVEF-left ventricular ejection fraction, ULV-upper limit of vulnerability, BMI-body mass index, CTR-cardiothoracic ratio, MI-myocardial infarction, CHF- congestive heart failure, ∆Vm- change in transmembrane potential, VF- ventricular fibrillation &  APD - action potential duration). Cutaway view of the ICD: Image reproduced with permission from St Jude medical. Inc.

At a macroscopic level the heart is viewed as a solid organ in the thorax whose electrical behavior can be altered by applying energy whereas at a microscopic level it can be viewed in the context of distribution and electrophysiological properties of various ion channels and gap junctions (Figure 1).

### Fibrillation and Defibrillation: role of electrical shocks - theoretical concepts

The two competing theories of ventricular fibrillation (VF) are the multiple wavelet hypothesis [11] and the mother rotor hypothesis [12]. The former, states that fibrillation is maintained by short-lived wavelets with constantly changing pathways. The wavelets may either be extinguished by encountering non conducting obstacles or get partially blocked causing fractionation into "daughter wavelets". When the tissue bulk involved exceeds a critical mass, enough daughter wavelets are constantly formed thus facilitating reentry and thereby sustaining fibrillation [13]. The mother rotor hypothesis states that a single stationary re-entrant circuit or a mother rotor located in the fastest activating region of the heart drives VF by giving rise to activation fronts that propagate and interact with anatomical and/or functional obstacles, causing fragmentation and new wavelet formation. Although these theories are distinct, there is some evidence that both can occur during different stages of VF [14]. The interplay of ionic currents, transmembrane potential (TMP) which is the electrical gradient between and intra and extracellular compartments, the composite resistance and capacitance of the  channels of the myocytes contribute to wave break, rotor stabilization and wave fragmentation thus promoting VF. To defibrillate, a shock must alter the TMP to a degree that it halts the VF wave fronts. However, if new wave fronts are created, then it can reinduce VF [15] thus increasing the DFT.

Two mechanisms have been proposed to explain how a shock can induce VF. The 'critical point hypothesis' based on theoretical considerations by Winfree [16] and experimental observations during electrical mapping experiments [17] espouses that reentry is formed at a critical point of the tissue refractoriness. This point is at which a critical degree of refractoriness of the tissue during the vulnerable period is intersected by a critical value of potential gradient created by the shock field. The value of the potential gradient at which reentry forms during the vulnerable period of a regular rhythm is approximately the same as the minimum potential gradient required throughout the ventricles for defibrillation [18]. This finding is consistent with the fact that the shock strength at the upper limit of vulnerability (ULV) is often used as a surrogate for the DFT. This supports the hypothesis that the mechanism by which a shock induces VF when given during the vulnerable period of cardiac cycle is similar to the mechanism by which a shock fails to defibrillate [19].

The second mechanism also utilizes the concept of reentry around a critical point; however, critical point is the pattern of shock induced virtual electrodes unlike the first mechanism [20,21]. These virtual electrodes are necessary to halt VF activation fronts present at the time of the shock; however post shock activation fronts formed in de-excited regions can favour reentry and hence VF thus raising DFT [20,21]. With increasing shock strength, the degree of hyperpolarization and depolarization increases in magnitude along with faster conduction velocity (CV) of the propagating activation front. When the CV is so rapid, the activation front reaches the tissue on the other side before it has had time to pass out of its refractory period, the activation front blocks without initiating reentry thus explaining the ULV mechanism [22].

## DFT: An Electrical Perspective

The battery and capacitor are the two integral components of an ICD that determine delivered energy. An ICD is designed to deliver shock energy to the critical mass of the left ventricle to stop the fibrillatory activation fronts. If it cannot be inhibited or if there is resumption of fibrillatory activation after a transient inhibition, higher shock energy is needed. There is no one simple electrical descriptor that quantifies defibrillation. Key parameters that influence the fibrillating heart is voltage and the duration for which it is applied. This is because the spatial derivative [23] of voltage is what interacts with the heart during a shock and duration is the time  a shock interacts with the fibrillating heart. The terms chronaxie and rheobase are properties of any excitable tissue. Rheobase is the minimum stimulus intensity needed to successfully defibrillate the heart, while chronaxie is the stimulus duration which corresponds to twice the rheobase. A larger amount of energy is needed for a shorter time and vice versa for effective defibrillation. On the other hand, impedance is the vector sum of all forces that oppose current flow in the device-lead-tissue circuit. Higher impedance affects the delivered energy thus increasing the DFT. Voltage is the electrical force that drives the electric current. Voltage as a function of time is the most relevant feature of electrical measurement in defibrillation and a minimum potential gradient is needed for successful defibrillation independent of the current value [18,24].

Capacitance is the ability of the capacitor in the device to hold charge. The capacitance should be large enough to raise the network voltage to its threshold and still hold enough charge to drive the network voltage back to zero during the second phase of the waveform. The cell membrane is charged up as the capacitor is discharged and if the energy is delivered beyond the peak transmembrane response, it will be wasted. Hence, the use of a high capacitance device may be counterproductive and may increase the DFT. A low capacitance device delivers higher voltage in a shorter time and makes it more efficient particularly in patients with higher resistance.

### Shock Energy, Shock vector and Shock waveforms

The shock energy is the energy delivered by the capacitor as a time function of the voltage discharge. The energy from any device is  *E = Voltage x Current x Time*. Delivered energy has no direct influence on defibrillation as it is just the difference between the energy left in the capacitor and the initial stored energy. The shock vector is the three dimensional orientation of the distribution of energy delivered by the device-coil system. Uniform distribution of energy encompassing the entire left ventricle is crucial. Hence the shock vector an important determinant of high DFT.

#### Shock waveforms:

In monophasic shocks, the polarity of each electrode remains same during the shock, whereas it reverses in biphasic shocks. The second phase of the biphasic shock removes the residual charge on the cells that were not captured and helps in returning the voltage response back to zero. This "burping" significantly diminishes the number of borderline stimulated cells  [25,26]. Biphasic shocks with reversed polarity are more effective than monophasic waveforms [27-36].  However, optimal duration of the two phases is critical and depends on the electrode impedance and the defibrillation capacitance [25,26,37,38]. Tilt is the percentage difference between the leading and trailing edge voltage of the biphasic waveform at the point where phase shifts.

### Effect of the Electrode Polarity

Polarity refers to the charge on either electrodes and is an important player in DFT especially with suboptimal shocks. In monophasic waveforms, the use of anodal defibrillation lead produces significantly lower DFT than cathodal defibrillation leads  [39,40]. During fibrillation, most of the myocytes are in the plateau phase and the cells near the anodal electrode are hyperpolarized. Cathodal shocks generate positive transmembrane potentials (TMP) which can activate cells in the virtual anode. Hence the virtual cathode launches wave fronts into the virtual anode [41]. In an anodal RV coil, the wave fronts from depolarized areas would go toward the coil and are merely extinguished. In a cathodal RV coil, wavefronts would be launched from there into the rest of the myocardium. This would be proarrhythmic leading to a higher DFT.

## DFT: An Electrophysiological Perspective

### Transmembrane potential: the key player

Successful defibrillation is realized through an electrical pulse that causes an alteration in the TMP of the myocytes in the critical mass of the myocardium. The cable model [42]  describes the generation of self propagating action potentials close to an electrode. However, it fails to explain the far field effects observed during defibrillation. The sawtooth model [43-45] and Bidomain model [46] on the other hand account for these. Both models incorporate active components like gap junctions, ion channels and membrane discontinuities. The Bidomain model is an extension of the one dimensional cable model where the extracellular and intracellular spaces are represented as single continuous domain extending in two or three dimensions. However, this is functionally separated by the cell membrane [46] which contributes anisotropic electrical conductivities [42].

### Insights Provided by the Bidomain Model to explain high DFT

Bidomain simulations have [47] demonstrated that the tissue response in terms of change in TMP (∆Vm) in the vicinity of a strong unipolar stimulus involved simultaneous occurrence of depolarizing and hyperpolarizing effects in close proximity. For example if an electrode is negative (a cathode), then the TMP becomes positive (is depolarized) directly under the electrode. However, when the tissue has unequal anisotropic ratios, there also exist regions of negative TMP (hyperpolarization) adjacent to the electrode along the fiber direction. The theoretical existence of virtual electrodes was contrary to the established view that tissue responses should only be depolarizing (cathodal stimulus) versus hyperpolarizing (anodal stimulus). The Bidomain model also explained the etiology of virtual electrode polarization (VEP) and its dependence on the cardiac tissue structure and the configuration of the applied field. VEP analysis demonstrated that both applied fields [48] and tissue structure [49,50] are  major determinants of the shape, location, polarity and intensity of the shock-induced VEP. The manner by which myocardial cells respond to a shock stimulus depends on its strength, polarity as well as the electrophysiological state of the cell at the time, the shock is delivered. Any pathological changes in the tissue such as scar periscar tissue will alter the VEP pattern and may influence the DFT.

### High DFT: Success or Failure of defibrillation - concepts from the Bidomain model

The pattern of VEP established in the 3D strongly depends on gross geometry and fiber orientation as well as on spatial nonuniformity of the applied field [49]. Studies have shown that, in all ranges of shock strengths and coupling intervals, the tissue in the LV free wall and septum are de-excited by the shock providing an excitable path for wave front propagation [51]. In contrast, RV free wall myocardium gets depolarized after the end of the shock. The geometry of the ventricles plays a vital role in the generation of post-shock arrhythmias. The ventricular wall asymmetry (thickness) manifests as a preferential location of post-shock excitable areas.  Simulations have demonstated that these areas are localized in the thick LV and septum and rather than thin RV [51]. Identifying these areas may be important for improving defibrillation efficacy since its eradication can specifically be targeted such as modified shock vector thus decreasing DFT [51].

 The upper limit of vulnerability (ULV) is defined as the shock strength applied during the peak of the T-wave above which fibrillation is noninducible. It is important to understand that this value is determined by applying energy to a heart in sinus rhythm whereas the DFT is determined by shocking a fibrillating heart. Even though studies have shown that the DFT correlates with the ULV [52,53], there may be physiological differences in the tissue whereby this relationship may falter [54]. Mazeh and Roth [22] published their study on the importance of myocardial fiber orientation to the mechanisms of the ULV. They found ULV is present if local heterogeneities are created by randomly placed fiber angles. When smooth fiber geometry is used in the model, reentry is induced regardless of the shock strength. Hence, local heterogeneities play an important role in the mechanism underlying the ULV. In the same context, Chen and Lin [55] have shown that temporal heterogeneity of repolarization at the time of shock and the differential responses of Ca_i_ to the shock could contribute to ventricular vulnerability and defibrillation. VEP contributes to post shock activation through its differential effects on the Ca_i_ transients [55]. The virtual anode increases the driving force of extracellular Ca entry via the already opened L-type Ca channel, which in turn increases Ca release from the sarcoplasmic reticulum mainly by hyperpolarizing the membrane potential on phase 3 of the action potential. In contrast, the virtual cathode produces the opposite effects on the Ca transients. Differential Ca transients at virtual anode and virtual cathode sites have been demonstrated both in cultured cells and in whole heart [56]. Biphasic shocks remove virtual electrode effects half way through the shock and hence can reduce Ca_i_ transients heterogeneity improving the efficacy of defibrillation [57].

## DFT: A Molecular Perspective

### Ion channels and their role in the maintenance and propagation of VF

Interaction between the voltage gated sodium current (I_Na_) and the inward rectifier current (I_K1_) is crucial for the control of normal cardiac excitability, stability and frequency of re-entry [14]. The I_K1_ stabilizes the resting membrane potential and is responsible for shaping the initial depolarization and final repolarization of the action potential [58]. During re-entry, a mismatch is established between the depolarizing current (mainly I_Na_) supplied by the wave front and the electrotonic (non action potential) current controlled by I_K1_. Hence a voltage gradient is created between the unexcited core and the neighboring excited cells which decreases the action potential duration (APD). The increased gradient supports a steeper rise in the local conduction velocity (CV) as one goes further from the core, leading to a faster and more stable rotor hence increasing the DFT. Vaquero [14] hypothesized that the inherent spatial heterogeneities in the distribution of the slow component of the delayed rectifier current (I_Ks_) can cause intermittent blocks (refractory areas) and spatially distributed wave-breaks which contribute to the propagation of fibrillation. This is due to the phenomenon of post repolarization refractoriness where myocyte activation failure can occur at stimulation frequencies at which I_Na_ has had enough time to recover from previous excitation. This is largely determined by the deactivation kinetics of I_Ks_  [59].

Prolongation of the cardiac action potential and the effective refractory period is a proven principle to prevent cardiac arrhythmias, especially in conditions where the action potential is shortened [60]. Pure K^+^ channel blockers or class III agents are known to decrease the DFT mainly due to lengthening of the refractory period [61].

### Cardiac Remodeling and ion channels: Effects on arrhythmogenesis and high DFT

 In heart disease, ion channel properties and gap junction distribution are responsive to changes in ionic fluxes, neurohumoral environment, and hemodynamic state [62]. In congestive heart failure, abnormalities in ion channels involved in automaticity ("funny" current I_f_) , early after depolarizations (EADs) (I_K1_, I_Ks_ and the transient outward K current - I_to_) and delayed afterdepolarizations (DADs) (late I_Na_, I_Na L_, increased Na^+^/Ca^2+^ exchanger function) promote arrhythmogenesis and may increase the DFT. In myocardial infarction, unidirectional block is favored by heterogenous K^+^ channel downregulation. Additionally connexin downregulation, decrease in I_Na_ and L type Ca^+2^ channel can slow down conduction thus leading to reentry. Ectopic complexes needed to intiate reentry and arrhythmogenesis are promoted by EADs and DADs [62].

### Ionic Mechanism of Defibrillation

The mechanism by which a shock accomplishes defibrillation is through an alteration in the TMP (∆Vm). Experimental studies using optical measurements have demonstrated that ∆Vm are strongly nonlinear during the plateau phase of the cardiac action potential [63]. This phenonmenon is directly applicable to situations when an ICD shock is delivered soon after VF onset, where the majority of the myocytes are in the plateau phase [63]. Two types of changes can occur here. Firstly, a negatively asymmetric ∆Vm where the hyperpolarization produced by one shock polarity is greater than the depolarization caused by the other shock polarity. The second is a nonmonotonic ∆Vm in which the polarization first increases but then decreases while a square wave stimulus is being given [64,65].

The effect of ∆Vm asymmetry might have important implications in the success or failure of defibrillation. Since most of the myocardium is in the plateau of the action potential during early VF, the effect of a defibrillation shock on ∆Vm should be asymmetric, with a predominantly negative ∆Vm.  As discussed in the Bidomain section, interaction between areas of hyperpolarization and depolarization can lead to reentry leading to defibrillation failure which has been studied in rabbit hearts [66].

The ∆Vm asymmetry with a larger shock-induced negative than positive ∆Vm reflects an outward shift in the balance of membrane currents. Inhibition of the typical outward currents I_K1_ and I_to_ do not reduce the ∆Vm in cell cultures indicating that none of these are responsible for the observed ∆Vm asymmetry  [64,67]. The asymmetric behavior of ∆Vm is reversed by the calcium channel blocker nifedipine in the cultured cell strands [68]. This effect of nifedipine on ∆Vm, suggests that ∆Vm asymmetry is caused by the outward flow of calcium current (I_Ca_) in the depolarized portions of the cell strands. Fast et al found that shocks cause transient decrease in Ca_i_^2+^ at sites of both negative and positive ∆Vm and that nifedipine eliminates the Ca_i_^2+^ decrease at the sites of positive ∆Vm  [66].  These results indicate that I_Ca_ flows in an outward direction in the areas of positive polarization, thus reducing the magnitude of positive ∆Vm during a shock [66].

The shock induced normotonic negative ∆Vm could be caused by the ionic currents which operate under large predominantly negative Vm, such as I_K1_, and I_f_  [69]. Inhibition of these currents however did not reverse the ∆Vm asymmetry. In cell cultures, Cheek and Fast demonstrated that the application of a series of shock strengths similar to those inducing a nonmonotonic ∆Vm in hyperpolarized regions, cause cell uptake of propidium iodide in the hyperpolarized region at the anodal side of cell strands but not in the cathodal region [70]. Hence the normotonic ∆Vm is caused by membrane electroporation rather than involvement of ion channels.

### Role of Gap Junctions in arrhythmogenesis and altering the defibrillation threshold

Gap junctions form the basis of the electrical syncitial properties of the heart  [71]. Connexin 43 represents the predominant gap junction protein in the human ventricle and its decreased level and the distribution can alter the arrhythmogenicity. Changes in gap junction distribution were first reported in patients with ischemic heart disease in the periinfarct zone and immunolabeling studies have confirmed this  [72].

Decreased connexin expression and phosphorylation contribute to conduction slowing in the failing heart which contributes to mechanical dysfunction, adverse cardiac remodeling and predispose to the generation of reentrant arrhythmias  [62]. Sims et al  [73] showed that regional infusion of the gap junction inhibitor heptanol increased the DFT by 33%. This is in contrast to a study by Qi  [74] where global infusion of gap junction blockers through isolated perfused rabbit hearts lowered the DFT considerably. These findings suggest that regional changes in the electrical properties of the heart are important in determining defibrillation efficacy  [73].

### Pharmacology, cellular electrophysiology and DFT

Cardiac and non cardiac medications (Table 1) through their actions on ion channels, electrolyte concentrations, neurohumoral modulation and intravascular volume can affect the defibrillation threshold. Sodium channel inhibition increases DFT with monophasic shocks but not biphasic shocks  [75,76]. This may be due to the differential effects on the ULV associated with monophasic shock. On the other hand, prolongation of cardiac repolarization by inhibition of K+ conductance has been shown to reduce the DFT  [77,78]. Drugs that prolong cardiac refractoriness reduce reentrant excitation  [79].  Ujhelyi  [78] reported that inhibition of outward K conductance with cesium chloride significantly reduced DFT.

Alterations in intracellular calcium levels have also been shown to influence DFT values. Flunarizine a Na^+^/Ca^2+^ exchange inhibitor is thought to improve defibrillation efficacy because it inhibits DADs, which are thought to cause the focal activation cycles arising after failed near-DFT shocks  [80].

The effect of anesthetic agents on DFT is very important as it is a silent participant in all DFT testing. Moerman et al reported that neither the anaesthetic technique nor the duration of anaesthesia was associated with significant changes in the defibrillation threshold  [81]. However Weinbroum et al showed in a randomized controlled trial that halothane, isoflurane, and fentanyl, when added to N_2_O/oxygen based general anesthesia increased the DFT while local anesthesia combined with intermittent small-dose propofol reduced the DFT  [82].

Propofol due to its excellent induction and emergence characteristics has become one of the most commonly used anesthetics during implantation. However a case report by Cohen illustrates the potential for an acute dose dependent rise in DFT with propofol  [83]. This information is pivotal as extra doses of propofol are commonly administered without proper titration of the depth of anesthesia during DFT testing.

A history of habitual cocaine use has also been shown to cause high DFT thereby necessitating a mandatory drug screen prior to ICD implantation  [84].

### Neurohumoral factors impacting DFT 

The role of the autonomic nervous system in the initiation and prevention of VF has been extensively studied, with most emphasis on the sympathetic arm. This assumes clinical importance since medications such as beta blockers are routinely used in patients who are at risk for VF. Although majority of the studies have concluded that catecholamines decrease DFT, there are few conflicting results   [85]  [-87]. Ruffy et al observed that beta-stimulation decreased DFT in the anesthetized dog heart. This effect was blocked with administration of propranolol before isoproteranol  [87], however,  Rattes et al  reported no change in DFT with administration of isoproteranol when DFT was  determined using a sequential pulse technique. Using a single pulse technique however isoproteranol produced a significant decrease in DFT  [85]. Thus, a potential advantage of the sequential pulse technique  is the stability of the  DFT despite the use of adrenergic agonists and antagonists.

In a separate study, pharmacological stimulation of the parasympathetic nervous system, either with muscarinic agonists or reflexively by enhancing baroreceptor response, was shown to have dual beneficial effects: increasing the ventricular fibrillation threshold, and decreasing the energy required for defibrillation  [86]. Evaluation of the effects of sympathetic stimulation by unloading the baroreceptors with nitroprusside infusion  no change in the DFT even with a mean systolic drop in blood pressure of 20 mmHg. Lerman et al hypothesized that the time dependent antiadrenergic effects of adenosine is responsible for the observed increase in DFT with prolonged episodes of VF  [54].  In a canine model, autonomic denervation abolished the effect of adenosine on the  DFT, suggesting that the effects of adenosine on the DFT is due to its antiadrenergic properties  [54].

## The Signs

### a. Pre implantation factors

A multitude of clinical, laboratory and echocardiographic factors that increase the risk of high DFT has been identified. The correlation of DFT with BMI and heart size is explicable given the requirement that an electric field of a certain threshold gradient (approximately 5 V/cm^2^) must be applied to >90% of the critical mass to terminate a ventricular fibrillation   [88]. Although some studies have shown that QRS duration is associated with higher DFT, it was found that the former was not an independent predictor of high DFT in multivariate analysis  [88-90]. In patients with hypertrophic cardiomyopathy, QRS duration is a predictor of high DFT  [91]. Additionally, several cardiac and non-cardiac medications including recreational drugs can increase the DFT [84] (Table 1).

### b. Implantation factors

Anesthetic agents are a silent participant in all DFT testing. Although the effect of anesthetics used during ICD implantation is controversial [81], its role cannot be ignored until further animal models reject the hypothesis. The location of the generator can also affect the DFT. It is often implanted taking into account the handedness and the comfort of the patient along with some pertinent medical conditions like an AV fistula, need for ipsilateral lung radiotherapy etc. It is therefore not uncommon to find patients who require ICD implantation on the right prepectoral region or even in the epigastrium [92,93], both of which may increase the DFT. The anatomic location of a "hot can" ICD generator (submuscular versus subcutaneous) influences the impedance to defibrillation current, however, it does not appear to alter the DFT [94]. As explained earlier, variations in the lead systems must also be taken into account. Shocks from an ICD are delivered from the coils of the leads that reach the generator by traversing through a critical portion of the myocardium enough to break the global wave of fibrillation. There can be a single or a dual coil incorporated into the lead. The distal coil sits well within the right ventricle while the proximal coil is designed to be positioned in the SVC. The actual position of these coils in a given patient affects the DFT. Typically, the ICD coil is placed at the apex of the right ventricle, but there is anecdotal evidence to show that a septal location close to the right ventricular outflow tract can reduce DFT [35]. Post implantation DFT changes can be time, drug and comorbidity dependent. Microdislodgement, increase in resistance due to fibrous tissue capsule formation, medications and clinical conditions may increase DFT after implantation.

## The Solutions

ICD implantation with current lead systems provides adequate safety margin in a vast majority of patients on initial implantation [95].  A high DFT can be noted during initial implantation, followup testing or device revision or generator change. Management of high DFT may require both non invasive and/or invasive management strategies.

### Non invasive strategies:

#### a. Identification of preventable causes of high DFT

Identification of preventable causes of high DFT such as medications, electrolyte abnormalities (hypomagnesemia, hypocalcemia, hyperkalemia) and acidosis etc is crucial. Heart failure status and medical therapy should be optimized prior to testing. Physicians should be vigilant for the development of hypoxia, hypercapnia or acidosis during anesthesia. The presence of any epicardial, intravascular or endocardial electrodes/patches increases the likelihood of high DFT. Additionally, a pneumothorax, or large pleural effusions may alter the shock impedance and the shock vector leading to high DFT.  Multiple testing should be avoided in patients with very low EF and borderline hemodynamic status. Moreover, prolonged anesthesia can worsen myocardial depression, hypotension, myocardial ischemia, and cause alteration in sympathetic tone leading to high DFT.  Timely recognition of these causes can prevent unnecessary interventions for high DFT.

#### b. System modification

Before DFT testing, it is imperative to confirm all the connections and measure the nominal impedance by low-voltage pulses. If anticipating a high DFT, then usage of high energy device is justified. Further system modification and advances in waveform optimization may reduce the number of difficult implants and instances of failed defibrillation [23]. St Jude Medical Systems incorporates programmable tilt, pulse width, polarity and shocking vector into some of their ICDs in order to noninvasively optimize the defibrillation shock.

#### c. Change of polarity

Available body of theoretical research and clinical outcome data is sufficient to conclude that in current ICDs the RV coil should be the anode. This configuration results in an average reduction of 16% in DFT compared to a cathodal RV coil [95]. Thus polarity reversal to reduce the DFT is useful only if initial testing was done using a cathodal RV coil. Clinical parameters have no value in predicting the effects of polarity changes on DFT. Hence, the efficacy of polarity reversal needs to be assessed in every patient  [96].

#### d. Tilt modification and fixed wave form duration

Biphasic waveforms of the new generation ICDs are truncated after the voltage has declined exponentially by 50-65% of its initial value. Tilt adjustment to the biphasic waveform may be tried in some patients with high DFT. Not all device manufacturers allow this option and no single tilt configuration is outright superior. Most devices have tilt as the nominal or the only choice for waveform duration. The use of millisecond optimized biphasic waveforms had lower DFTs compared with conventional tilt-based waveforms in patients with high DFT [23].

#### e. Medications

Sotalol is reported to decrease DFT hence it can potentially be used in the management of patients with high DFT. A study by Simon et al showed that Dofetilide significantly reduces DFT and prevents the need for more complex lead systems [97]. There are no data available regarding the effect of the newer multichannel antiarrhythmic drugs on DFT.

### Invasive factors:

#### a. Use of high output ICD device

Although it is advisable to use high output devices at the time of implantation in patients where high DFT is anticipated, longer charge time, quicker battery drain and increased risk of post shock electromechanical dissociation should be anticipated. Older age, lower EF, worse NYHA functional class, recent use of amiodarone (within previous six weeks) and right sided pre-pectoral implants are known to have high DFT and may benefit from high energy devices.

#### b. Altering the shock vector

RV lead repositioning, manipulation of the SVC coil, addition of subcutaneous arrays, additional coil implantation in the azygous vein, cornary sinus and epicardial space are the other techniques designed to  alter the  shock vector. The RV lead should be positioned at the apex with achievement of sensing > 5mV and pacing < 1V thresholds is crucial. Proximal relocation of the RV coil results in higher DFTs. However, moving the RV lead closer to the interventricular septum as well as RVOT positioning will improve the DFT [98-100]. With the adoption of dual coil single - lead systems as standard practice, the ability to manipulate and optimally position the proximal coil in SVC has become limited. High SVC and left brachiocephalic positioning appear to be better than low SVC- right atrial (RA) positioning of the proximal coil. The latter configuration creates a suboptimal vector, shunting the current away from the LV into the SVC through the RA leading to a higher DFT. With a tuned waveform, the addition of an SVC coil to the shocking pathway reduces DFTs, although this difference is smaller compared to other invasive measures [101].

Adding an SVC coil to an active can configuration decreases the defibrillation energy requirement but paradoxically increases peak current suggesting that the vector is worsened with an SVC coil, however this effect is offset by a large reduction in shock impedance.  Hence patients with high DFT who already have low impedance can benefit by removal of SVC coil and changing to unipolar configuration. This can be accomplished electronically in some devices. Addition of a brachiocephalic/left subclavian coil can be useful in patients who have single coil lead or those with dual coil leads where the proximal coil is in the low SVC - RA junction. The addition of an azygous vein or coronary sinus (CS) coil improves the shock vector thus lowering the DFT; however, the stability of coil in the CS is disputed. Subcutaneous array implantation is the most efficacious strategy for managing high DFT although it is an invasive, painful procedure requiring general anesthesia.

#### c. Using a lower capacitance ICD

Theoretical models of defibrillation show that the optimal capacitance is inversely related to inter-electrode resistance  [38,102]. The benefits of reduced capacitance are more evident in patients with high resistance [103]. Shortening the first phase of the biphasic waveform makes it closer to the defibrillation chronaxie. In addition, the second phase of the waveform can be made closer to passive membrane time constant required for optimum membrane discharge as previously mentioned [95].

## Conclusion

Although technological advancements have improved the delivery of shock energy, the challenge of high DFT will be more common with the increasing number of ICD implantations. High DFT is the result of a complex interplay between molecular, electrical, mechanical, anatomical, neurohumoral and pharmacological factors. Hence a clear understanding of the mechanism and a scientific step-wise approach to manage patients with high DFT forms the crux of the solution. The recognition of at-risk patients for high DFT is essentially based on history and clinical information and the implanting physician needs to be cognizant of the fact that an awareness of these factors helps in appropriate planning before implantation.

## Figures and Tables

**Table 1:**
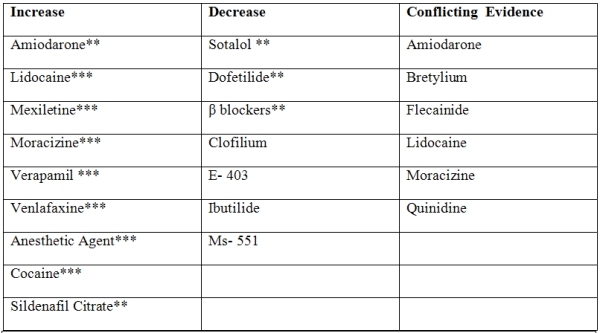
Various Medications that Influence the Defibrillation threshold

*Data derived from multiple randomized clinical trials or meta-analyses. **Data derived from a single randomized trial or nonrandomized studies. ***Only consensus opinion of experts, case studies or standard-of-care.
